# Alloparental Support and Infant Psychomotor Developmental Delay

**DOI:** 10.1007/s12110-024-09468-4

**Published:** 2024-02-14

**Authors:** David Waynforth

**Affiliations:** School of Medicine, Faculty of Health Sciences, Bond University, Gold Coast, QLD 4229 Australia

**Keywords:** Grandmothers, Alloparenting, Allomothers, Inclusive fitness, Fathers, Paternal care

## Abstract

Receiving social support from community and extended family has been typical for mothers with infants in human societies past and present. In non-industrialised contexts, infants of mothers with extended family support often have better health and higher survival through the vulnerable infant period, and hence shared infant care has a clear fitness benefit. However, there is scant evidence that these benefits continue in industrialised contexts. Better infant health and development with allocare support would indicate continued evolutionary selection for allocare. The research reported here used multiple logistic regression analysis to test whether a lack of family and other social support for mothers was associated with an increased risk of developmental delay in 9-month-old infants in the UK Millennium Cohort (analysis sample size, 15,696 infants). Extended family-based childcare during work hours and more maternal time spent with friends were the most influential kin and social support variables: infants of mothers with kin-based childcare versus all other childcare arrangements had a lower risk of developmental delay (OR = 0.61, 95% CIs: 0.46–0.82). Infants of mothers who spent no time with friends when compared with those who saw friends every day had double the odds of delay. Greater paternal involvement in infant care was associated with a lower odds of developmental delay. In conclusion, shared care of infants and social support for mothers may influence fitness-related traits in industrialised societies rather than being factors that influenced selection only in the past and in societies which retain close kin networks and a strong local community focus.

**Supplementary Information:**

The online version contains supplementary material available at 10.1007/s12110-024-09468-4.

## Introduction

Compared with many other mammal infants, human infants are highly dependent on parental care, with a long period of dependency and substantial time and energetic burdens on mothers (Zeveloff & Boyce, 1982; Isler & van Schaik, 2012). The parental burden is typically not borne by mothers alone but is shared with family and community members (Coall & Hertwig, 2010; Hrdy, 2009; Isler & van Schaik, 2012; Kramer & Veile, 2018; Piperata, 2009; Sear, 2016; Wilson, 1975). Help can consist of direct infant care, helping behaviours such as performing domestic activities, and emotional support to lessen the stress on mothers. There is evidence that the children of women who are able to mitigate the costs of maternal care by using alloparental assistance experience better health and have higher survival (Sear & Mace, 2007; Waynforth, 2020]. The purpose of this research was to test the hypothesis that infants born to mothers with social and family support living in the contemporary UK experience a broader range of benefits than those regarding morbidity and mortality. Specifically, the hypothesis was that these infants will be less likely to show delay in psychomotor development. Psychomotor delay has been demonstrated to have long-term sequelae affecting social and economic success, such as memory problems, lower IQ and poorer social functioning (Cantwell & Baker, 1977; Feldman et al., 2000; Johnson et al., 1999; Perna & Loughan, 2012). These sequelae are likely to affect fitness.

### Kin Selection and Cooperative Breeding in Humans

For the majority of parents in industrialised economies, childcare is an economic arrangement between parents and paid carers, with additional support from family if they happen to be present. But this does not reflect typical childcare patterns outside of the industrialised world. In hunter-gatherer and small-scale agricultural societies, mothers usually perform around half of an infant or young child’s direct care, fathers provide relatively little care, and family members perform much of the remainder. Grandmothers and elder siblings are the most important allocarers in many societies (Crittenden & Marlowe, 2008; Ivey, 2000; Kramer & Veile, 2018; Turke, 1988).

Kin selection is an explanation for why parents should prefer family members as additional childcare providers, and why their care should lead to improved child outcomes compared with care from non-relatives (Hamilton, 1964). Evolutionary kin selection theory implies that genetic relatives should be more motivated to provide high quality, stimulating child care since they will benefit genetically if differences in care lead to advantages for the child. In evolutionary terms, fitness is an individual’s genetic contribution to future generations. Any behaviours which influence fitness will also influence representation of the genes that underlie the behaviours in future generations. Altruistic actions directed towards genetic relatives, while costly to the actor, can benefit and spread copies of the actor’s genes present in their genetic relatives (Hamilton, 1964). This should be particularly true of grandparental care, where the actor may be past reproductive age themselves yet can increase genetic contribution to future generations by caring for young relatives (Hawkes et al., 1998). Hence kin selection explains the evolution of intergenerational and other cooperation between genetic relatives.

### Types of Social and Community Support

Non-kin can aid mothers both directly through childcare as well as indirectly, such as by helping with household tasks, providing advice and sharing personal experiences, or simply by listening and helping new mothers feel support and companionship. Support from non-kin may come from other mothers and community members and is commonly given as childcare from older children (Gray, 1995; Piperata, 2009). Social support from non-relatives in small-scale communities appears to be mutually beneficial, involving reciprocal altruistic acts of support, care, and sharing of work burdens without the actors necessarily keeping score or accounts of each act of generosity (Silk, 2003).

### Effects of Kin-Based Childcare and Support on Child Health and Development

Grandparental support or presence for mothers of young children is often associated with higher infant and child survival in nations with lower infant survival (Sear & Mace, 2007). But in contexts with high infant survival the relationship between family support and infant health outcomes is mixed. In the UK Millennium Cohort Study, grandparental infant care is associated with a lower risk of infant health problems leading to hospitalisation, including for infectious disease (Waynforth, 2020), but other studies have not shown consistent evidence of health effects of grandparenting, with the exception of grandparental involvement being associated with weight gain in infants (reviewed in Pulgaron et al., 2016).

In non-human cooperative breeders, the presence and number of alloparents is associated with faster growth, development, and time from hatching to fledging (Burkart et al., 2009; Komdeur, 1994; Ridley, 2007). Alloparenting effects on cognitive development may be particularly important in species which are prosocial with complex social structures (Burkart et al., 2009). In humans, to date there have been relatively few studies published on grandparental care and children’s cognitive development in any society. Sadruddin et al.’s (2019) systematic review identified five high-quality studies. Their findings suggest a complex pattern in which the associations between grandparenting and child development are in different directions in high versus low socioeconomic groups. Much of the evidence shows disadvantages of having a grandparent as the main custodial caregiver, particularly when combined with low socioeconomic position.

### Effects of Community-Based Social Support on Child Health and Development

There is some evidence from cohort studies that social support to mothers is beneficial for infant development. In the UK ALSPAC longitudinal cohort study, social support was found to be positively associated with child development both directly and via mothers’ church support networks (Shaver et al., 2020). There are three areas of evidence linking support from non-kin to child development which include mechanisms and pathways. First, development can be influenced via effects on maternal energy budgets, allowing more energy to be allocated to the infant. In support of this link, social support for mothers has been shown to lessen the energetic burden of lactation in nursing mothers and the amount of labour that mothers perform (Bove et al., 2002; Piperata, 2009; Snell-Rood & Snell-Rood, 2020). Second, contact, and touch in particular, from community members has positive effects on infant neurodevelopment via hormonal and enzymatic triggers (reviewed in Mrljak et al., 2022; Racine et al., 2019). Third, there are indirect benefits to infants from social support to mothers via effects of social support on maternal mental health, which in turn benefits maternal parenting behaviours and child development (Balaji et al., 2007; Kingston et al., 2012; Shaver et al., 2020).

### Study Prediction

The hypothesis tested here was that kin-based allocare and support, and measures of social support from non-relatives, will be associated with a reduced likelihood of developmental delay in infancy in the contemporary UK.

## Methods

### Population and Sample

The UK Millennium Cohort Study (henceforth MCS) is an ongoing longitudinal study of 18,827 infants born in the United Kingdom from September 2000 to August 2001. Mothers were identified using Universal Child Benefit records and NHS Health Visitors. They were interviewed about many aspects of their pregnancy, the birth of the cohort member, health, work, and parenting. The MCS was created as a multidisciplinary cohort study for analysis by health, social, and economic scientists (Ketende & Jones, 2011). Here, data were analysed using the first survey of the cohort, which took place when the infants were around 9 months old. Oversampling was carried out to compensate for lower response rates from a few demographic groups which had occurred in prior UK national cohort studies: families from economically disadvantaged areas, ethnic minorities, and less-populated regions of the UK were oversampled to better reflect the demographics of the UK. A cohort profile is available providing detail about the sample and sampling methods (Connelly & Platt, 2014).

### Dependent Variable

The MCS questionnaire items relevant to developmental delay closely resembled the commonly used Ages and Stages inventory items capturing variation in cognitive and motor skill domains (Goldsmith, 2021). A parent or main caregiver reported developmental progress on 12 questions related to cognitive and motor skills development. Responses to the 12 questions were on three-point scales, coded as “1” for the infant frequently demonstrates the developmental milestone, “2” for sometimes, and “3” for the infant has not yet demonstrated the milestone. The 12 items were sits up, smiles, stands up while holding on, puts hands together, grabs objects, holds small objects, passes a toy, walks a few steps, gives toy, waves bye-bye, extends arms, and nods for yes. The responses were summed into a single score. Because development scores should be highly associated with an infant’s exact age at the time of interview, centred residuals were created to produce a development score that is independent of age. Residual centring was carried out by regressing infants’ ages in days on their development score and saving the standardised residuals as a variable. Since the aim of the study was to capture delayed development rather than development as a continuous variable, a binary variable with the cut point at two standard deviations below the mean age-adjusted score was created as the study’s dependent variable.

### Independent Variables

Variation in family and social support was represented by six measures: The frequency the mother reported seeing her mother (coded as lives with = 0 and sees her every day = 1 to never seeing her either because she is not alive, is inaccessible, or because the mother has not maintained contact = 5). Family-based childcare as the usual arrangement during work hours was coded as a binary variable. Whether the mother believes that family would help if financial problems occurred was reported on a five-point scale from strongly agree = 1 to strongly disagree = 5. Mothers reported having other mothers to talk to and spending time with friends using a five-point scale ranging from every day = 1 to never = 5. The MCS additionally included the number of people who attended the birth, reported by the mother.

Covariates for the regression analysis were chosen on the basis of being established as predictors of infant health and development in previous studies, and factors with potential to interact with family support: for example, mothers who receive more support from their partner may seek less support from family and friends (Alio et al., 2009; Andreev, 2000; Ketterlinus et al., 1990; McIntire et al., 1999; Nikiéma et al., 2007; Singletary, 2021; Waynforth, 2022). Covariates included were the mother’s age; number of siblings (full and half) in the household; socioeconomic status (McClements equivalised income); birthweight; infant sex; and paternal presence in the household. Ethnicity was included by creating binary variables for the maternal ethnic backgrounds that were represented frequently enough to avoid empty cells in the regression. These were South Asian (Pakistani, Indian and Bangladeshi), African, and Afro-Caribbean. Infants in poorer health may be more likely to show developmental delay and have different care arrangements. To account for this in the statistical models, the infant’s number of hospital admissions (maternally reported) was included as a covariate. Formal daycare in daycare facilities was included as a dummy variable.

The MCS interview with fathers included questions about how often the father performs childcare behaviours. Each was answered on a six-point scale from “never” to “more than once a day.” The activities included were the father caring for the infant on his own, getting up in the night when the baby cries, changing nappies, and feeding. These were summed to create a single scale with equal weighting for each paternal activity.

### Data Analysis

The analysis used multiple logistic regression as a null hypothesis significance test of whether family and social support are associated with a reduced likelihood of developmental delay. All six allocare and social support variables were entered into the logistic regression together so that the result for each is statistically adjusted for the other allocare and social support variables. Because these variables might be expected to have substantial correlations with each other, variance inflation due to multicollinearity was explored using VIF and with bivariate correlations. Alpha was set at *p* < .05. Additional logistic regression models were run to handle multicollinear variables, and subgroup analyses were performed for father-present and father-absent households separately to determine whether results for the allocare and social support variables were different for these household types. Due to the lower interview response rate for fathers in the MCS, an additional logistic regression analysis was carried out for cases with missing paternal care data.

Two-way plots were generated to determine whether any of the continuous or scale predictors had nonlinear associations with probability of developmental delay, and attempts were made to linearise relationships that were observed not to be linear. All analyses were carried out in Stata 16.

## Results

### Descriptive Statistics

Table 1 shows descriptive statistics for all variables included in the logistic regression models. The 12 measures of developmental progress contained some items that almost all infants had been observed to do by nine months, such as smiling, whereas at the other end of the spectrum, few were walking at nine months. Of the family and social support variables, 17% of mothers listed a kin-allocarer (most often the infant’s maternal grandmother) as the main daytime infant care provider. On average, mothers reported seeing their mother and seeing friends once or twice a week.

**Table 1:** Descriptive statistics

	Variable	Obs	Mean (SD) or n for categorical data	Min–Max
Outcome	Age-adjusted development below − 2SD of sum of 12 main caregiver responses listed below.	18,432	Y = 3638, *N* = 14,794	0–1
Child development measures	Smiles	18,432	1.01 (0.08)	1–3
	Sits up	18,432	1.07 (0.32)	1–3
	Stands up holding on	18,432	1.48 (0.78)	1–3
	Puts hands together	18,432	1.21 (0.53)	1–3
	Grabs objects	18,432	1.01 (0.12)	1–3
	Holds small objects	18,432	1.15 (0.45)	1–3
	Passes a toy	18,432	1.07 (0.30)	1–3
	Walks a few steps	18,432	2.81 (0.52)	1–3
	Gives a toy	18,432	1.52 (0.72)	1–3
	Waves bye-bye	18,432	1.91 (0.84)	1–3
	Extends arms	18,432	1.21 (0.50)	1–3
	Nods for yes	18,432	2.72 (0.62)	1–3
Family & social support	Frequency mother sees her mother	18,544	3.28 (2.35)	0–8
	Mother has other parents to talk to	17,805	2.10 (1.02)	1–5
	Family would help if financial problems	17,803	1.75 (0.97)	1–5
	Frequency mother reports spending time with friends	18,527	2.96 (0.97)	1–5
	Number of people who attended birth	18,432	1.12 (0.50)	0–4
	Family-based infant care in work hours	18,387	Y = 3126, *N* = 15,261	1–2
Statistical control variables	Equivalised household income	18,432	296.83 (217.1)	14.31–1250.78
	Birthweight (kg)	18,382	3.34 (0.59)	0.391–7.229
	Infant’s number of hospitalisations	18,422	1.63 (1.99)	0–50
	Infant’s sex	18,432	M = 8976, F = 9456	1–2
	Mother’s age (years)	18,426	28.1 (5.95)	13–51
	Paternal involvement score: how much help father is.	16,255	10.20 (5.87)	1–21
	Paternal presence in the household	18,406	Y = 17,191, Sometimes = 317, *N* = 898	1–3
	Number of siblings of infant in household	18,552	0.94 (1.08)	0–9
	Formal daycare in work hours	18,432	Y = 2123, *N* = 16,309	
	Maternal ethnic origin: South Asia	18,402	Y = 1736, *N* = 16.666	1–2
	Maternal ethnic origin: Africa	18,402	Y = 377, *N* = 18,205	1–2
	Maternal ethnic origin: Caribbean	18,402	Y = 263, *N* = 18,139	1–2

All variables are from maternal or main care provider interviews with the exception of paternal care scores, which were taken from interviews with the father

### Correlations Between Allocare, Paternal, and Maternal Support Variables

Table 2 displays correlations for associations between paternal presence and childcare with the allocare variables. One of the main concerns was that paternal support would be highly associated with allocare. For example, it appeared plausible that families with fathers who performed a large amount of childcare would have less need for other support, and therefore these would be highly negatively correlated. As can be seen in Table 2, this expectation was not consistently supported. For example, higher paternal care scores were associated with using daytime family-based childcare (for example, by grandparents). Kin-based allocare was highly correlated with how often mothers reported seeing their mother (*r* = − 0.23), which was in turn highly associated with maternal expectations that they would receive financial help from their parents if they require it (*r* = 0.24).

**Table 2:** Bivariate correlations between paternal presence in the household, paternal childcare involvement and the allocare variables

	Variables	(1)	(2)	(3)	(4)	(5)	(6)	(7)	(8)
(1)	Father presence in household (Yes = 1, Sometimes = 2, No = 3)	1.000							
(2)	Paternal care score (1 = none to 21 = high support)	−.321**	1.000						
(3)	Frequency sees own mother (0 = every day to 8 = never)	−.032**	.074**	1.000					
(4)	Family would help if financial problems (agree = 1 to disagree = 5)	.052**	−.048**	.239**	1.000				
(5)	Other parents to talk to (every day = 1 to never = 5)	.044**	−.090**	.001	.265**	1.000			
(6)	Frequency mother spends time with friends (every day = 1 to never = 5)	−.035**	.054**	.009	.047**	.179**	1.000		
(7)	Family-based infant care during work hours (No = 0, Yes = 1)	−.052**	.083**	−.228**	−.091**	−.032**	.058**	1.000	
(8)	Number of people who attended birth	.014	−.029**	−.147**	−.067**	−.009	−.048**	.038**	1.000

* *p* < .05, ** *p* < .01

### Regression Results

Birthweight and equivalised income had nonlinear relationships with developmental delay. Birthweight was successfully transformed using its reciprocal, and the direction of the odds ratio reported in the results table reflects this. Income had a nonlinear relationship with delay that was not successfully handled using arithmetic transformation. It was retained in the logistic regression model.

The regression model fit and r-squared statistics indicated a statistically satisfactory model (pseudo *r*^2^ = 0.11), and there were no influential outliers present (max. Cook’s Distance < 0.02). VIF values were smaller than expected given the high correlations observed in Table 2: the largest VIF score was 1.43 (mother’s age).

**Fig. 1:**
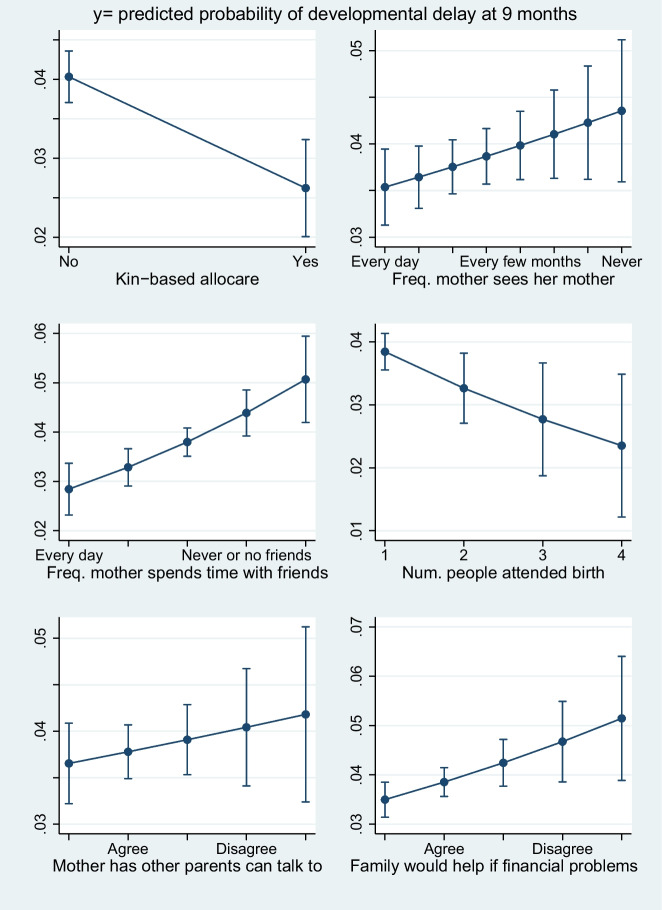
Margins plots showing results of the logistic regression analysis shown in Table 3. Predictive margins and 95% CIs are displayed for the family and social support variables

Table 3 displays regression results for the model with covariates, and Fig. 1 displays estimated marginal means plots of the family and social support variables in the logistic regression so their relationships with probability of developmental delay can be visually assessed (with the other variables in the model held constant). Each of the six support measures was associated with developmental delay (see Table 3 and the direction of the relationships shown in Fig. 1). Four of the six allocare and maternal support variables were statistically significantly associated with a reduced risk of developmental delay. The frequency that mothers reported seeing their mother and having other parents to talk to were not statistically significant in the full model displayed in Table 3. However, these nonsignificant results appeared to be due to multicollinearity: Table 4 displays a logistic regression model with kin-based allocare removed. In its absence, infants of mothers who see their mother more frequently had a lower odds of developmental delay (OR = 1.06, 95% CI: 1.02–1.10, *p* < .001). Similarly, having other parents to talk to was significantly associated with a lower odds of developmental delay with time spent with friends and financial help from parents removed from the model (OR = 1.10, 95% CI: 1.02–1.18, *p* < .02, see Table 5). Tables S1–S6 in the Electronic Supplementary Materials (ESM) display the allocare and social support variables entered into logistic regression models without the other five allocare and support variables entered. All six variables were statistically significant predictors of delay in these models.

**Table 3:** Logistic regression results

						95% Confidence Interval	
Predictor	Estimate	SE	*Z*	*p*	Odds Ratio	Lower	Upper
Intercept	−4.49	0.46	−9.79	< .001	0.01	0.00	0.03
1/Birthweight (*z*-scores)	0.56	0.03	18.35	< .001	1.75	1.65	1.86
Infant sex (1 = M, 2 = F)	−0.57	0.09	−6.27	< .001	0.57	0.47	0.68
Infant number of health problems	0.06	0.02	4.03	< .001	1.06	1.03	1.10
Infant’s number of siblings	0.09	0.04	2.13	.034	1.09	1.01	1.19
Maternal age	0.02	0.01	2.69	.007	1.02	1.01	1.04
South Asia	−0.33	0.17	−1.92	.055	0.72	0.52	1.01
Caribbean	−0.85	0.46	−1.85	.064	0.43	0.17	1.05
Africa	−0.98	0.43	−2.30	.022	0.37	0.16	0.87
Income (*z*-scores)	0.01	0.05	0.27	.787	1.01	0.92	1.12
Father present in household (yes = 1, sometimes = 2, no = 3)	−0.16	0.11	−1.51	.131	0.85	0.69	1.05
Paternal care score (1 = none to 21 = highest)	−0.02	0.01	−2.04	.041	0.98	0.97	1.00
Daycare facility is main weekday care arrangement	−0.39	0.16	−2.47	.014	0.68	0.50	0.92
Kin-based allocare is main weekday care arrangement	−0.49	0.15	−3.36	< .001	0.61	0.46	0.82
Frequency mother sees her mother (0 = daily to 8 = never)	0.03	0.02	1.66	.097	1.03	0.99	1.08
Family would help if money problems (agree = 1 to disagree = 5)	0.11	0.04	2.51	.012	1.12	1.02	1.22
Other parents to talk to (1 = daily to 5 = never)	0.04	0.04	0.87	.385	1.04	0.95	1.13
Frequency mother has time with friends (1 = often to 5 = never)	0.16	0.05	3.50	< .001	1.18	1.08	1.29
Number of people who attended birth	−0.19	0.09	−1.99	.046	0.83	0.69	1.00

Dependent variable is presence of developmental delay (adjusted for infant age in days). *N* = 15,696. Model coefficients: Developmental delay at least 2SD below mean. (pseudo *r*^2^ = 0.11)

**Table 4:** Logistic regression model result with kin-based allocare excluded to examine multicollinearity with the frequency mother sees her mother, demonstrating that the frequency that mothers reported seeing their mother is statistically significant with kin-based allocare during work hours excluded

						95% Confidence Interval	
Predictor	Estimate	SE	*Z*	*p*	Odds Ratio	Upper	Lower
Intercept	−5.09	0.39	−13.03	< .001	0.01	0.00	0.01
1/Birthweight (*z*-scores)	0.54	0.03	19.59	< .001	1.72	1.63	1.82
Infant sex (1 = M, 2 = F)	−0.57	0.09	−6.65	< .001	0.57	0.48	0.67
Infant number of health problems	0.06	0.02	3.70	< .001	1.06	1.03	1.09
Infant’s number of siblings	0.09	0.04	2.35	.019	1.10	1.02	1.19
Maternal age	0.02	0.01	2.38	.017	1.02	1.00	1.04
South Asia	−0.34	0.15	−2.23	.026	0.71	0.53	0.96
Caribbean	−0.86	0.44	−1.96	.050	0.42	0.18	1.00
Africa	−0.86	0.37	−2.31	.021	0.42	0.20	0.88
Income (*z*-scores)	−0.04	0.05	−0.78	.438	0.96	0.87	1.06
Daycare facility is main weekday care arrangement	−0.31	0.15	−2.05	.041	0.74	0.55	0.99
Number of people at birth	−0.26	0.09	−2.90	.004	0.77	0.65	0.92
Other parents to talk to (1 = daily to 5 = never)	0.05	0.04	1.09	.277	1.05	0.96	1.13
Frequency mother sees her mother (0 = daily to 8 = never)	0.06	0.02	3.18	.001	1.06	1.02	1.10
Father present in household (yes = 1, sometimes = 2, no = 3)	−0.11	0.10	−1.07	.284	0.90	0.74	1.09
Family would help if money problems (agree = 1 to 5 = disagree)	0.09	0.04	2.17	.030	1.09	1.01	1.19
Frequency mother has time with friends (1 = often to 5 = never)	0.14	0.04	3.11	.002	1.15	1.05	1.25

Model Coefficients: Developmental delay at least 2SD below mean

Estimates represent the log odds of “Developmental delay at least 2SD below mean = 1” vs. “Developmental delay at least 2SD below mean = 0”

**Table 5:** Logistic regression model result examining multicollinearity of maternal reports of having other parents to talk to, with time with friends (*r* = 0.18) and financial help from parents (*r* = 0.27) excluded

						95% Confidence interval	
Predictor	Estimate	SE	*Z*	*p*	Odds Ratio	Upper	Lower
Intercept	−3.92	0.41	−9.46	< .001	0.02	0.01	0.04
1/Birthweight (*z*-scores)	0.54	0.03	19.37	< .001	1.72	1.63	1.82
Infant sex (1 = M, 2 = F)	−0.57	0.09	−6.72	< .001	0.56	0.48	0.67
Infant’s number of health problems	0.05	0.02	3.49	< .001	1.05	1.02	1.09
Infant’s number of siblings	0.08	0.04	1.93	.054	1.08	1.00	1.17
Maternal age	0.02	0.01	2.80	.005	1.02	1.01	1.04
South Asia	−0.28	0.15	−1.88	.060	0.75	0.56	1.01
Caribbean	−0.82	0.44	−1.86	.063	0.44	0.19	1.05
Africa	−0.76	0.37	−2.05	.040	0.47	0.22	0.97
Income (*z*-scores)	−0.02	0.05	−0.45	.656	0.98	0.89	1.08
Daycare facility is main weekday care arrangement	−0.41	0.15	−2.69	.007	0.67	0.49	0.89
Number of people who attended birth	−0.28	0.09	−3.18	.001	0.75	0.63	0.90
Kin-based allocare is main weekday care arrangement	−0.51	0.14	−3.71	< .001	0.60	0.46	0.79
Other parents to talk to (1 = daily to 5 = never)	0.09	0.04	2.36	.018	1.10	1.02	1.18
Frequency mother sees her mother (0 = daily to 8 = never)	0.05	0.02	2.75	.006	1.05	1.01	1.09
Father present in household (yes = 1, sometimes = 2, no = 3)	−0.12	0.10	−1.22	.222	0.88	0.72	1.08

With these measures excluded, having other parents to talk to is statistically significantly associated with a lower odds of developmental delay. Model Coefficients: Developmental delay at least 2SD below mean

Estimates represent the log odds of “Developmental delay at least 2SD below mean = 1” vs. “Developmental delay at least 2SD below mean = 0”

Subgroup analysis results for father-present and father-absent households are shown in Tables S7 and S8. Kin and social support did not clearly appear to have different influences on the odds of infant developmental delay in father-absent and father-present households. Similarly, in households missing paternal care information (*n* = 2257, see Table S9), the odds ratios for the six allocare and social support variables do not differ substantially from the main model results shown in Table 3.

## Discussion & Conclusions

### Main Findings

The evolutionary theoretical paradigm underlying this research argues that kin-based allocare and other support for mothers is typical of and important for reproductive success in our species and is a key aspect of human sociality (Coall & Hertwig, 2010; Hrdy, 2009; Sear, 2016). The results reported here were consistent with this: 17% of infants were cared for during weekdays by biological kin, most commonly maternal grandmothers. After handling the expected problems with entering correlated variables into a regression model, all six measures of kin and social support were associated with a reduced probability of having developmental delay by nine months. Using the regression model results for all support variables together, an infant with a mother with no family or social support had around an eight times increased odds of developmental delay when compared with a mother with the maximum possible level of family and social support. Put into the context of developmental delay as a relatively rare event, this equates for a male infant to close to a 10% probability of delay with no social or family support versus a 1% probability of delay with the highest levels of support.

### Paternal Influences

It was expected that lack of paternal involvement would be associated both with developmental delay and with maternal social and family support, hence the inclusion of paternal support as control variables in the regression models. More paternal involvement in childcare was associated with a lower likelihood of delay, but unexpectedly, father-absent infants did not have an increased likelihood of delay. An interesting pattern of associations can be seen in Table 2, where more paternal childcare was associated with mothers spending less time with their friends, and with less interaction with the infant’s maternal grandmother. Paternal absence from the household was associated with mothers seeing the infant’s maternal grandmother more often, but also with less daytime weekday allocare. When added to logistic regression models, interaction terms between paternal and allocare variables were not statistically significant (models not shown).

### Effects of the Statistical Control Variables

Of the statistical control variables, low birthweight had a sizeable effect on the odds of developmental delay occurring. Infants with older mothers, male infants and those who had more hospital admissions in their first nine months of life had an increased odds of developmental delay. Infants with more siblings had an increased odds of delay, which may reflect maternal depletion. Income and ethnicity were not statistically significant in the main logistic regression model, with the exception of maternal African origin, which was associated with a reduced odds of delay. It should be noted that the effects of ethnic origin were greater without kin-based allocare during work hours in the regression model. This suggests multicollinearity such that the protective effects of being born to a South Asian, African, or Caribbean origin may be in part due to kin-based allocare in these households (see Tables 3 and 4). Infants in formal daycare arrangements had a lower odds of developmental delay, with a statistical effect size similar to that observed for kin-based informal daycare. This suggests that delay may be most likely to occur in households in which the mother cares for her child without the help of family or formal daycare for the infant.

### Comparisons with Existing Studies

This analysis found protective effects of kin and social support measures for developmental delay, but the overall pattern of findings in other studies has been mixed. Sadruddin et al.’s (2019) systematic review of effects of grandparental involvement in childrearing on children’s health and development did not show consistent evidence of beneficial effects for infants in industrialised contexts. Indeed, some studies included in the systematic review found that grandmaternal care is associated with worse infant health rather than better outcomes. Some of these findings may occur when grandparental care is custodial rather than provided as extra childcare help for mothers. Several relevant studies have been published since Sadruddin et al.’s review, and these have also shown mixed results. For example, Singletary (2021) found that infant social and motor skill developmental milestones were reached earlier in children whose mothers had the highest levels of alloparental support. Morita et al.’s (2021) path analysis study found only an indirect association via effects of grandmaternal support on mothers’ mental health. This finding raises another unresolved question: what specific activities are grandmothers, other allocarers, and maternal support-providers doing that makes a difference to child development? The results reported here may align with Morita et al.’s, and with research linking maternal mental health with child development in that having alloparental support, feeling that your parents would rescue you financially if you were in financial difficulties, and spending time with friends each could affect maternal mental health and be a reason for their associations with a reduced likelihood of developmental delay (Balaji et al., 2007; Kingston et al., 2012; Shaver et al., 2020).

### Study Limitations

The UK Millennium Cohort Study data allowed analysis with a level of statistical power that is difficult to achieve in studies without very substantial financial backing. But this came at a cost in that the family support variables were not created specifically to test this study’s hypothesis and were from interview data rather than collected by methods which directly measure social support. In addition, it is unclear how and if friends and other mothers are supportive, or if these relationships are in part competitive rather than purely supportive. Methods which directly measure social support and disentangle it from interactions which are not supportive would be preferable. For example, one ongoing study has taken an experimental approach, offering social support to a cohort of mothers and comparing infant outcomes with those of infants born to mothers not enrolled in the support programme (Waijungbah Jarjums Project, 2021). Although detailed statistical analysis has not been published at the time of writing, this experimental approach suggests that social support has a larger effect on infant health than the findings for the MCS cohort reported here.

The analysis carried out here focussed on developmental delay when the infants were 9 months old. The MCS is longitudinal, and further analysis could determine whether infants with developmental delay continued to have developmental delay once they were in a school setting, and whether kin-based allocare and social support to mothers remain important later in child development.

### Supplementary Information

Below is the link to the electronic supplementary material.

ESM 1 (PDF 343 KB)

## Data Availability

The raw data used in this study are available free of charge by registering with the UK Data Service: https://ukdataservice.ac.uk/.
