# Supplementary material for: Alloparental Support and Infant Psychomotor Developmental Delay
Source: Hum Nat. 2024 Feb 14;35(1):43–62. doi: 10.1007/s12110-024-09468-4 (PMC11052766; doi:10.1007/s12110-024-09468-4)
Supplement: Supplementary file 1 — (PDF 343 KB) [file 12110_2024_9468_MOESM1_ESM.pdf]

Supplementary material

Title

Alloparental support and infant psychomotor developmental delay.

Journal

Human Nature 35(1), 2024. https://doi.org/10.1007/s12110-024-09468-4

Author

David Waynforth

School of Medicine, Faculty of Health Sciences

Bond University, Gold Coast, Queensland 4229, Australia

Email: dwaynfor@bond.edu.au

Table S1. Logistic regression results for kin-based allocare is main weekday care arrangement, excluding the five other family and social support variables.

Table S2. Logistic regression results for frequency mother sees her mother, excluding the five other family and social support variables.

Table S3. Logistic regression results for family would help if financial problems, excluding the five other family and social support variables.

Table S4. Logistic regression results for mother has other parents to talk to, excluding the five other family and social support variables.

Table S5. Logistic regression results for frequency mother has time with friends, excluding the five other family and social support variables.

Table S6. Logistic regression results for number of people who attended birth, excluding the five other family and social support variables.

Table S7. Logistic regression results for father present households only.

Table S8. Logistic regression results for father absent and sometimes present households only.

Table S9. Logistic regression results for cases missing paternal information.

Table S1. Model Coefficients - Developmental delay at least 2SD below mean

Predictor	Estimate	SE	Z	p	Odds ratio	95% Confidence Interval	
						Lower	Upper
Intercept	-4.11	0.35	-11.76	< .001	0.02	0.01	0.03
1/Birthweight (z-scores)	0.55	0.03	19.92	< .001	1.74	1.65	1.84
Infant sex, 1=M, 2=F	-0.56	0.08	-6.72	< .001	0.57	0.49	0.67
Infant number of health problems	0.05	0.01	3.25	0.001	1.05	1.02	1.08
Infant's number of siblings	0.10	0.04	2.75	0.006	1.11	1.03	1.20
Maternal age	0.03	0.01	3.63	< .001	1.03	1.01	1.04
South Asia	-0.14	0.13	-1.03	0.305	0.87	0.67	1.13
Caribbean	-0.89	0.44	-2.01	0.044	0.41	0.17	0.98
Africa	-0.67	0.35	-1.94	0.053	0.51	0.26	1.01
Income (z-scores)	-0.02	0.05	-0.41	0.684	0.98	0.89	1.08
Daycare facility is main weekday care arrangement	-0.42	0.15	-2.75	0.006	0.66	0.49	0.89
Kin-based allocare is main weekday care arrangement	-0.60	0.13	-4.49	< .001	0.55	0.42	0.71

Note. Estimates represent the log odds of "Developmental delay at least 2SD below mean = 1" vs. "Developmental delay at least 2SD below mean = 0"

Table S2. Model Coefficients - Developmental delay at least 2SD below mean

Predictor	Estimate	SE	Z	p	Odds ratio	95% Confidence Interval	
						Lower	Upper
Intercept	-4.97	0.30	-16.40	< .001	0.01	0.00	0.01
1/Birthweight (z-scores)	0.55	0.03	20.21	< .001	1.74	1.65	1.83
Infant sex, 1=M, 2=F	-0.56	0.08	-6.74	< .001	0.57	0.49	0.67
Infant number of health problems	0.05	0.01	3.33	< .001	1.05	1.02	1.08
Infant's number of siblings	0.12	0.04	3.17	0.002	1.13	1.05	1.21
Maternal age	0.02	0.01	2.75	0.006	1.02	1.01	1.04
South Asia	-0.22	0.14	-1.60	0.109	0.80	0.61	1.05
Caribbean	-0.94	0.44	-2.12	0.034	0.39	0.17	0.93
Africa	-0.85	0.35	-2.41	0.016	0.43	0.22	0.85
Income (z-scores)	-0.05	0.05	-1.06	0.288	0.95	0.86	1.04
Daycare facility is main weekday care arrangement	-0.30	0.15	-2.02	0.043	0.74	0.55	0.99
Freq. mother sees her mother, 0=daily to 8=never	0.07	0.02	3.94	< .001	1.07	1.03	1.11

Note. Estimates represent the log odds of "Developmental delay at least 2SD below mean = 1" vs. "Developmental delay at least 2SD below mean = 0"

Table S3. Model Coefficients - Developmental delay at least 2SD below mean

						95% Confidence Interval	
**Predictor**	**Estimate**	**SE**	**Z**	**p**	**Odds ratio**	**Lower**	**Upper**
Intercept	-5.20	0.31	-16.57	< .001	0.01	0.00	0.01
1/Birthweight (z-scores)	0.55	0.03	19.75	< .001	1.73	1.64	1.82
Infant sex, 1=M, 2=F	-0.56	0.08	-6.63	< .001	0.57	0.48	0.67
Infant number of health problems	0.05	0.02	3.58	< .001	1.06	1.02	1.09
Infant's number of siblings	0.10	0.04	2.66	0.008	1.11	1.03	1.20
Maternal age	0.03	0.01	3.55	< .001	1.03	1.01	1.04
South Asia	-0.17	0.15	-1.14	0.254	0.85	0.63	1.13
Caribbean	-0.84	0.44	-1.91	0.056	0.43	0.18	1.02
Africa	-0.69	0.37	-1.85	0.064	0.50	0.24	1.04
Income (z-scores)	-0.04	0.05	-0.76	0.446	0.96	0.87	1.06
Daycare facility is main weekday care arrangement	-0.27	0.15	-1.83	0.067	0.76	0.57	1.02
Family would help if money problems, agree=1, to 5	0.14	0.04	3.64	< .001	1.15	1.07	1.24

Note. Estimates represent the log odds of "Developmental delay at least 2SD below mean = 1" vs. "Developmental delay at least 2SD below mean = 0"

Table S4. Model Coefficients - Developmental delay at least 2SD below mean

Predictor	Estimate	SE	Z	p	Odds ratio	95% Confidence Interval	
						Lower	Upper
Intercept	-5.21	0.32	-16.22	< .001	0.01	0.00	0.01
1/Birthweight (z-scores)	0.54	0.03	19.63	< .001	1.72	1.63	1.82
Infant sex, 1=M, 2=F	-0.56	0.08	-6.63	< .001	0.57	0.48	0.67
Infant number of health problems	0.05	0.02	3.51	< .001	1.06	1.02	1.09
Infant's number of siblings	0.11	0.04	2.88	0.004	1.12	1.04	1.21
Maternal age	0.03	0.01	3.83	< .001	1.03	1.02	1.05
South Asia	-0.14	0.15	-0.98	0.329	0.87	0.65	1.16
Caribbean	-0.84	0.44	-1.90	0.058	0.43	0.18	1.03
Africa	-0.63	0.37	-1.68	0.092	0.54	0.26	1.11
Income (z-scores)	-0.04	0.05	-0.78	0.437	0.96	0.87	1.06
Daycare facility is main weekday care arrangement	-0.27	0.15	-1.83	0.067	0.76	0.57	1.02
Other parents to talk to, 1=daily to 5=never	0.10	0.04	2.49	0.013	1.10	1.02	1.19

Note. Estimates represent the log odds of "Developmental delay at least 2SD below mean = 1" vs. "Developmental delay at least 2SD below mean = 0"

Table S5. Model Coefficients - Developmental delay at least 2SD below mean

Predictor	Estimate	SE	Z	p	Odds ratio	95% Confidence Interval	
						Lower	Upper
Intercept	-5.35	0.33	-16.40	< .001	0.00	0.00	0.01
1/Birthweight (z-scores)	0.55	0.03	19.97	< .001	1.73	1.64	1.82
Infant sex, 1=M, 2=F	-0.55	0.08	-6.60	< .001	0.58	0.49	0.68
Infant number of health problems	0.05	0.01	3.44	< .001	1.05	1.02	1.08
Infant's number of siblings	0.13	0.04	3.55	< .001	1.14	1.06	1.23
Maternal age	0.03	0.01	3.32	< .001	1.03	1.01	1.04
South Asia	-0.18	0.14	-1.33	0.183	0.84	0.64	1.09
Caribbean	-0.92	0.44	-2.10	0.036	0.40	0.17	0.94
Africa	-0.71	0.35	-2.03	0.042	0.49	0.25	0.97
Income (z-scores)	-0.04	0.05	-0.89	0.374	0.96	0.87	1.05
Daycare facility is main weekday care arrangement	-0.29	0.15	-1.96	0.050	0.75	0.56	1.00
Freq. mother has time with friends, 1=often to 5=never	0.15	0.04	3.59	< .001	1.16	1.07	1.26

Note. Estimates represent the log odds of "Developmental delay at least 2SD below mean = 1" vs. "Developmental delay at least 2SD below mean = 0"

Table S6. Model Coefficients - Developmental delay at least 2SD below mean

						95% Confidence Interval	
**Predictor**	**Estimate**	**SE**	**Z**	**p**	**Odds ratio**	**Lower**	**Upper**
Intercept	-4.43	0.33	-13.45	< .001	0.01	0.01	0.02
1/Birthweight (z-scores)	0.54	0.03	19.96	< .001	1.72	1.63	1.82
Infant sex, 1=M, 2=F	-0.56	0.08	-6.72	< .001	0.57	0.49	0.67
Infant number of health problems	0.05	0.01	3.33	< .001	1.05	1.02	1.08
Infant's number of siblings	0.12	0.04	3.11	0.002	1.12	1.04	1.21
Maternal age	0.02	0.01	2.94	0.003	1.02	1.01	1.04
South Asia	-0.13	0.13	-0.99	0.324	0.88	0.67	1.14
Caribbean	-0.86	0.44	-1.97	0.048	0.42	0.18	0.99
Africa	-0.72	0.35	-2.04	0.041	0.49	0.25	0.97
Income (z-scores)	-0.05	0.05	-0.99	0.323	0.95	0.87	1.05
Daycare facility is main weekday care arrangement	-0.28	0.15	-1.90	0.058	0.75	0.56	1.01
Number of people at birth	-0.33	0.09	-3.78	< .001	0.72	0.61	0.85

Note. Estimates represent the log odds of "Developmental delay at least 2SD below mean = 1" vs. "Developmental delay at least 2SD below mean = 0"

Table S7. Father present in household cases only

Model Coefficients - Developmental delay at least 2SD below mean

Predictor	Estimate	SE	Z	p	Odds ratio	95% Confidence Interval	
						Lower	Upper
Intercept	-4.25	0.43	-9.76	< .001	0.01	0.01	0.03
1/Birthweight (z-scores)	0.55	0.03	17.37	< .001	1.73	1.63	1.84
Infant sex, 1=M, 2=F	-0.57	0.09	-6.08	< .001	0.57	0.47	0.68
Infant number of health problems	0.06	0.02	4.00	< .001	1.06	1.03	1.10
Infant's number of siblings	0.08	0.04	1.84	0.066	1.08	0.99	1.18
Maternal age	0.03	0.01	2.81	0.005	1.03	1.01	1.04
South Asia	-0.28	0.17	-1.63	0.103	0.75	0.54	1.06
Caribbean	-1.16	0.57	-2.05	0.041	0.31	0.10	0.95
Africa	-0.70	0.43	-1.62	0.105	0.50	0.21	1.16
Income (z-scores)	-0.00	0.05	-0.08	0.936	1.00	0.90	1.10
Paternal care score, 1=none, to 21=highest	-0.02	0.01	-1.93	0.054	0.98	0.97	1.00
Daycare facility is main weekday care arrangement	-0.35	0.16	-2.21	0.027	0.70	0.52	0.96
Kin-based allocare is main weekday care arrangement	-0.46	0.15	-3.12	0.002	0.63	0.47	0.84
Freq. mother sees her mother, 0=daily to 8=never	0.04	0.02	1.87	0.061	1.04	1.00	1.08
Family would help if money problems, agree=1, to disagree=5	0.11	0.05	2.38	0.017	1.11	1.02	1.22
Other parents to talk to, 1=daily to 5=never	0.06	0.04	1.44	0.150	1.07	0.98	1.16
Number of people at birth	-0.25	0.10	-2.52	0.012	0.78	0.64	0.95

Note. Estimates represent the log odds of "Developmental delay at least 2SD below mean = 1" vs. "Developmental delay at least 2SD below mean = 0"

Table S8. Father sometimes present or absent in household cases only (1215 individuals) Note: African families excluded due to too few father absent households.

Model Coefficients - Developmental delay at least 2SD below mean

						95% Confidence Interval	
Predictor	Estimate	SE	Z	p	Odds ratio	Lower	Upper
Intercept	-4.11	2.01	-2.05	0.041	0.02	0.00	0.84
1/Birthweight (z-scores)	0.70	0.11	6.24	< .001	2.01	1.61	2.50
Infant sex, 1=M, 2=F	-0.71	0.37	-1.90	0.058	0.49	0.24	1.02
Infant number of health problems	-0.02	0.09	-0.19	0.848	0.98	0.83	1.17
Infant's number of siblings	0.24	0.16	1.49	0.136	1.27	0.93	1.72
Maternal age	-0.00	0.04	-0.05	0.963	1.00	0.93	1.07
South Asia	0.25	0.64	0.39	0.694	1.28	0.37	4.46
Caribbean	-0.02	0.84	-0.03	0.978	0.98	0.19	5.12
Income (z-scores)	0.51	0.43	1.17	0.243	1.66	0.71	3.88
Daycare facility is main weekday care arrangement	-0.99	1.11	-0.90	0.369	0.37	0.04	3.23
Kin-based allocare is main weekday care arrangement	-0.14	0.65	-0.21	0.832	0.87	0.24	3.14
Freq. mother sees her mother, 0=daily to 8=never	-0.11	0.08	-1.28	0.200	0.90	0.76	1.06
Family would help if money problems, agree=1, to 5	0.09	0.16	0.58	0.565	1.10	0.80	1.52
Other parents to talk to, 1=daily to 5=never	0.23	0.16	1.45	0.148	1.26	0.92	1.74
Number of people at birth	0.09	0.29	0.31	0.754	1.10	0.62	1.95

Note. Estimates represent the log odds of "Developmental delay at least 2SD below mean = 1" vs. "Developmental delay at least 2SD below mean = 0"

Table S9. Cases missing paternal care information (n=2257)

Model Coefficients - Developmental delay at least 2SD below mean

Predictor	Estimate	SE	Z	p	Odds ratio	95% Confidence Interval	
						Lower	Upper
Intercept	-2.15	1.24	-1.73	0.084	0.12	0.01	1.33
1/Birthweight (z-scores)	0.46	0.07	6.16	< .001	1.58	1.37	1.83
Infant sex, 1=M, 2=F	-0.56	0.25	-2.20	0.028	0.57	0.35	0.94
Infant number of health problems	-0.02	0.06	-0.35	0.724	0.98	0.87	1.10
Infant's number of siblings	-0.05	0.12	-0.39	0.698	0.95	0.75	1.21
Maternal age	0.02	0.02	0.99	0.322	1.02	0.98	1.07
South Asia	-0.40	0.35	-1.16	0.245	0.67	0.34	1.32
Caribbean	-1.03	1.24	-0.83	0.408	0.36	0.03	4.09
Income (z-scores)	-0.08	0.16	-0.52	0.606	0.92	0.67	1.27
Daycare facility is main weekday care arrangement	-0.86	0.62	-1.39	0.166	0.42	0.13	1.43
Kin-based allocare is main weekday care arrangement	-0.77	0.44	-1.76	0.078	0.46	0.20	1.09
Freq. mother sees her mother, 0=daily to 8=never	0.12	0.05	2.37	0.018	1.13	1.02	1.25
Family would help if money problems, agree=1, to 5	-0.11	0.14	-0.79	0.429	0.90	0.68	1.18
Other parents to talk to, 1=daily to 5=never	0.01	0.12	0.08	0.936	1.01	0.79	1.29
Number of people at birth	-0.84	0.28	-3.03	0.002	0.43	0.25	0.74

Note. Estimates represent the log odds of "Developmental delay at least 2SD below mean = 1" vs. "Developmental delay at least 2SD below mean = 0"
